# Lytic Bacteriophage PZL-Ah152 as Biocontrol Measures Against Lethal *Aeromonas hydrophila* Without Distorting Gut Microbiota

**DOI:** 10.3389/fmicb.2022.898961

**Published:** 2022-07-12

**Authors:** Chao Feng, Kaixiang Jia, Teng Chi, Shuaimin Chen, Huabo Yu, Liang Zhang, Sayed Haidar Abbas Raza, Ahmed Mohajja Alshammari, Shuang Liang, Zishan Zhu, Tingxuan Li, Yanling Qi, Xiaofeng Shan, Aidong Qian, Dongxing Zhang, Lei Zhang, Wuwen Sun

**Affiliations:** ^1^College of Animal Science and Technology, Jilin Agricultural University, Jilin, China; ^2^Institute of Agricultural Resources and Environment, Jilin Academy of Agricultural Sciences, Changchun, China; ^3^College of Animal Science and Technology, Northwest A&F University, Xianyang, China; ^4^Department of Biology, College of Science, University of Hail, Ha’il, Saudi Arabia

**Keywords:** *Aeromonas hydrophila*, phage therapy, phage genome, phage safety, gut microbiota

## Abstract

Phage therapy is an alternative approach to overcome the problem of multidrug resistance in bacteria. In this study, a bacteriophage named PZL-Ah152, which infects *Aeromonas hydrophila*, was isolated from sewage, and its biological characteristics and genome were studied. The genome contained 54 putative coding sequences and lacked known putative virulence factors, so it could be applied to phage therapy. Therefore, we performed a study to (i) investigate the efficacy of PZL-Ah152 in reducing the abundance of pathogenic *A. hydrophila* strain 152 in experimentally infected crucian carps, (ii) evaluate the safety of 12 consecutive days of intraperitoneal phage injection in crucian carps, and (iii) determine how bacteriophages impact the normal gut microbiota. The *in vivo* and *in vitro* results indicated that the phage could effectively eliminate *A. hydrophila*. Administering PZL-Ah152 (2 × 10^9^ PFU) could effectively protect the fish (2 × 10^8^ CFU/carp). Furthermore, a 12-day consecutive injection of PZL-Ah152 did not cause significant adverse effects in the main organs of the treated animals. We also found that members of the genus *Aeromonas* could enter and colonize the gut. The phage PZL-Ah152 reduced the number of colonies of the genus *Aeromonas.* However, no significant changes were observed in α-diversity and β-diversity parameters, which suggested that the consumed phage had little effect on the gut microbiota. All the results illustrated that PZL-Ah152 could be a new therapeutic method for infections caused by *A. hydrophila.*

## Introduction

*Aeromonas* spp., frequently associated with severe infections in cultured fish species, is the most common bacterium found in freshwater habitats (Liu X. et al., 2020). *Aeromonas hydrophila* infects various freshwater fish species and also causes severe diseases in humans, such as pneumonia, empyema, and peritonitis (Citterio and Francesca, 2015; Tsujimoto et al., 2019). Antibiotics are still the best way to treat *A. hydrophila* infection. However, excessive antibiotic administration had made *A. hydrophila* strains antibiotic-resistant (Zhu et al., 2020). Therefore, new antimicrobial therapies urgently need to be developed.

Bacteriophages (or phages) are abundant in nature and can target and destroy pathogenic bacteria. At present, phages are gradually accepted as alternatives/complements to antibiotic therapy (Danis-Wlodarczyk et al., 2021) and have been successfully used in many bacterial pathogens causing diseases in animals and humans (Jikia et al., 2005; Jun et al., 2013). Some medical institutions are performing phase II clinical trials of bacteriophage (Duplessis and Biswas, 2020). Moreover, some phages can penetrate the biofilms. The research on *A. hydrophila* phage is also increasing. The phages G65 and Y81 showed a considerable bactericidal effect and potential in preventing the formation of *A. hydrophila* biofilms, and the phages G65, W3, and N21 were able to scavenge mature biofilms effectively (Liu J. et al., 2020). To treat *A. hydrophila* infection, phage mixture therapy was established based on the analysis of the genomic sequences and biological characteristics of vB_AHAp_PZL-AH8 and vB_AHAp_PZL-AH1 (Yu et al., 2022). The results also showed that phage therapy was a good way to inhibit the production of phage-resistant strains. Some studies have shown that phage pAh6-C could treat the diseases caused by *A. hydrophila* infection in carps (Yun et al., 2019). Bacteriophages pAh-1 and Akh-2 also had good therapeutic effects in the treatment of *A. hydrophila* infection in zebrafish and loach (Easwaran et al., 2017; Akmal et al., 2020).

The predominant bacterial taxa in the fish gut, such as *Proteobacteria*, *Fusobacteriota*, and *Firmicutes*, play important roles in promoting cellulose decomposition and polysaccharide fermentation in the gut (Kostic et al., 2013). In a zebrafish model, changes in the intestinal flora directly affected fish growth and development, as well as the morphology of the intestinal mucosa and the regeneration of intestinal epithelial cells (Rawls et al., 2004). Studies have shown that the addition of phages to the mice enteritis infection model could not only kill target cells but also produce a cascade effect on other bacteria through bacterial interactions without disrupting the homeostasis of the intestinal microbiota (Hsu et al., 2019). These abilities of phages reflect their strong application potential (Febvre et al., 2019).

In this study, we isolated a bacteriophage (PZL-Ah152) that infected a pathogenic strain of multidrug-resistant (MDR) *A. hydrophila* 152. After sequencing and analyzing the PZL-Ah152 genome, we discovered a lack of bacterial virulence- or lysogenesis-related ORFs, which suggested that this phage was eligible for therapy. A phage therapy experiment in *A. hydrophila*-infected crucian carps was performed based on the phage’s ability to kill the target bacteria. In addition, there are no reports showing whether phage administration affects the commensal *A. hydrophila* that colonizes the gastrointestinal tract of fish.

## Materials and Methods

### Fish and Ethics Statement

In this study, crucian carp (average weight 35 ± 1 g) specimens obtained from a fish farm were used. All experiments were performed rigorously under the Regulations for the Administration of Affairs Concerning Experimental Animals that were issued by the State Council of the People’s Republic of China (1988.11.1), as well as in accordance with the guidelines of the Animal Welfare and Research Ethics Committee at Jilin Agriculture University (JLAU08201409).

### Bacterial Strains and Growth Conditions

*Aeromonas hydrophila* 152 was used for phage isolation, and 39 additional *Aeromonas* strains (including 21 *A. hydrophila* and 18 *A. veronii*) were used for host range analysis (Table 1). All the bacterial strains were cultured in LB broth at 37°C.

**TABLE 1:** Bactericidal spectrum of PZL-Ah152.

Organism	Strain name	Spot testing results	Organism	Strain name	Spot testing results
*A. hydrophila*	36	**–**	*A. veronii*	1	**–**
*A. hydrophila*	54	**–**	*A. veronii*	3	**–**
*A. hydrophila*	64	**–**	*A. veronii*	4	**–**
*A. hydrophila*	68	**–**	*A. veronii*	5	**–**
*A. hydrophila*	69	**+**	*A. veronii*	6	**+**
*A. hydrophila*	87	**+**	*A. veronii*	7	**–**
*A. hydrophila*	93	**–**	*A. veronii*	9	**–**
*A. hydrophila*	103	**+**	*A. veronii*	11	**–**
*A. hydrophila*	107	**+**	*A. veronii*	20	**–**
*A. hydrophila*	119	**+**	*A. veronii*	21	**–**
*A. hydrophila*	138	**–**	*A. veronii*	47	**–**
*A. hydrophila*	142	**+**	*A. veronii*	77	**–**
*A. hydrophila*	143	**–**	*A. veronii*	85	**–**
*A. hydrophila*	147	**–**	*A. veronii*	115	**–**
*A. hydrophila*	148	**–**	*A. veronii*	155	**–**
*A. hydrophila*	150	**+**	*A. veronii*	520	**–**
*A. hydrophila*	166	**–**	*A. veronii*	QXF0711B	**–**
*A. hydrophila*	183	**–**	*A. veronii*	TH0426	**–**
*A. hydrophila*	1021	**–**			
*A. hydrophila*	BSK	**–**			
*A. hydrophila*	TPS	**+**			

*“+” Bacteria can be cleaved by bacteriophage; “–” Bacteria cannot be cleaved by bacteriophage.*

### Phage Isolation and Host Range Testing

*Aeromonas hydrophila* 152 was adopted for phage propagation in the current study. PZL-Ah152 was isolated from sewage systems in Changchun, China. Phage isolation, plaque assays, and spot tests were conducted according to previously reported methods (Gu et al., 2011; Hyman, 2019). A measure of 5 μL of phage (1.0 × 10^7^ PFU/ml) was spotted on double-layered plates containing different *A. hydrophila* strains for the detection of the host range. After overnight incubation, the zone of lysis of the susceptible host was observed.

### Biological Characteristics of the Phage

One-step growth curve determination was performed according to a previously described method with slight modifications (Kropinski, 2017). Briefly, *A. hydrophila* 152 was incubated in an LB medium until the logarithmic phase (OD600 nm = 0.5), and the culture was infected with bacteriophage PZL-Ah152 with an MOI of 0.1 and kept at 37°C for 5 min for adsorption. The cultures were centrifuged to remove the unabsorbed bacteriophage, and the precipitate was resuspended in 10 ml of LB. A total of 0.1 ml of culture was transferred to 9.9 ml of LB. A 10-fold serial dilution of the mixture was carried out two times and incubated at 37°C. Then, 0.1 ml of the sample was collected every 5 min for PFU determination by plaque determination on a double-layer LB plate. The average burst size was quantified as the difference between the final and the initial phage titers divided by the initial phage titer.

For pH stability tests, the effect of varying pH on a 100 μL PZL-Ah152 (1.0 × 10^7^ PFU/ml) was determined; for this purpose, the bacteriophage was cultured in LB broth adjusted to pH 2–13 for 1 h, and aliquots were taken to measure phage titers at different pH values. For thermal stability tests, 2 ml of bacteriophage (1.0 × 10^7^ PFU/ml) was incubated at 30°C, 40°C, 50°C, 60°C, 70°C, and 80°C. Then, 100 μL aliquot samples were collected at intervals of 10 min. Moreover, we incubated aliquot samples of phage PZL-Ah152 suspensions at 4°C for 1 year. All tests were performed in triplicate.

### Electron Microscopy and Protein Analyses

Bacteriophage was condensed and modified as previously studied (Gong et al., 2016). Bacteriophage PZL-Ah152 was amplified in 800 ml LB and centrifuged at 8,000 × *g* for 15 min at 4°C to remove bacterial debris. Phage particles were precipitated by the addition of 10% polyethylene glycol 8000 and 1 M NaCl and subsequently disbanded in 5 ml PBS. CsCl (1.45, 1.50, 1.70 g/ml) was added to the supernatant and centrifuged at 120,000 × *g* for 3 h at 4°C. The phage band was harvested and dialyze.

Phage morphology was observed by negative phosphotungstic acid staining. A 20 μL aliquot of the concentrated PZL-Ah152 suspension was applied to copper grids stained negatively with 1% phosphotungstic acid (pH 7) for 30 s. Electron microscopy of JEOL (JEM-1400, Japan) was operated at 80 kV. Afterward, 10 μL of phage particles purified by CsCl density-gradient centrifugation were mixed with a gel electrophoresis sample buffer, boiled for 10 min, and subjected to SDS–polyacrylamide gel electrophoresis (8–16% gradient). Protein bands were visualized by using Instant Blue staining (Expedeon Protein Solutions Ltd., Cambridge, United Kingdom).

### Phage DNA Extraction, Sequencing, and Annotation

Phages (≥ 10^11^ PFU/ml) were filtered through a 0.22 μm filter. The genomic DNA of the phage was extracted using a Universal Phage Genomic DNA Extraction Kit (Knogen, Guangzhou, China). The extracted phage DNA was sequenced at Sangon Biotech (Shanghai, China) by using an Illumina HiSeq 2500 sequencing system. Open reading frames (ORFs) were predicted with BLAST and GeneMarkS.

### Phage PZL-Ah152 Killing Assay *in vitro*

*Aeromonas hydrophila* 152 mixed with PZL-Ah152 at MOIs of 1, 0.1, and 0.01 was cultured in tubes filled with LB broth (1 × 10^8^ CFU/ml). After incubation at 37°C for 1, 2, 3, 6, 9, and 12 h, the cell survival rate was detected. A bacterial culture without the phage was used as the control, and a colony count was carried out.

### Phage Treatment of Infected Fish

For the safety assay, PZL-Ah152 was intraperitoneally administered to wild-type crucian carps (1.0 × 10^10^ PFU/ml, 200 μL/fish) for 12 consecutive days, while the control group was exposed to the same volume of PBS. Tissue sampling (*n* = 3) was performed on days 1, 4, and 12 postexposure. Liver, spleen, kidney, gut, and gill tissues were surgically removed to conduct hematoxylin and eosin (H&E) staining analysis and qRT-PCR. The mRNA expression levels of TGF-β, IFN-γ, TNF-α, IL-1β, and IL-10 were quantified by qPCR. The primers used for the immunity-related genes and β-actin, which was considered a housekeeping gene, are listed in Table 2.

**TABLE 2:** Sequences and conditions of the primers used in RT-PCR analysis.

Gene	Nucleotide Sequence (5′-3′)	Annealing Temp (°C)	NCBI Accession No.
IL-1β	F: AACTGATGACCCGAATGGAAAC	55	AY340959.1
	R: CACCTTCTCCCAGTCGTCAAA		
TNF-α	F: TTATGTCGGTGCGGCCTTC	55	AY427649.1
	R: AGGTCTTTCCGTTGTCGCTTT		
IL-10	F: GGAACGATGGGCAGATCAA	60	AY887900.1
	R: AACTGAAGGGGAAGGGGAAG		
IFN-γ	F: AACAGTCGGGTGTCGCAAG	60	EU909368.1
	R: TCAGCAAACATACTCCCCA		
TGF-β	F: CTGGCTCTTGCTCTTTCGTCT	60	EU086521
	R: AAGGATGGGCAGTGGGTCT		
β-actin	F: CAAGATGATGGTGTGCCAAGTG	58	AF025305
	R: TCTGTCTCCGGCACGAAGTA		

To determine the minimal lethal dose (MLD), six crucian carps in each experimental group were injected intraperitoneally (i.p.) with *A. hydrophila* 152 (at concentrations of 1 × 10^6^, 1 × 10^7^,1 × 10^8^, 1 × 10^9^, and 1 × 10^10^CFU/ml, 100 μL/fish). No bacterial inoculation was performed in the control group, and 2 × MLD was used as the infective inoculum.

Crucian carps infected with 2 × MLD (2 × 10^9^ CFU/ml, 100 μL/fish) of *A. hydrophila* 152 were treated with PZL-Ah152 at different concentrations (1 × 10^8^, 1 × 10^9^, and 1 × 10^10^ PFU/ml, 100 μL/fish) (*n* = 20 in the respective groups) after 1 h. The crucian carps were infected with 2 × MLD (2 × 10^9^ CFU/ml, 100 μL/fish) of *A. hydrophila* 152, and 12 h and 24 h later, the crucian carps were treated with PZL-Ah152 (1 × 10^10^ PFU/ml, 200 μL/fish, *n* = 20 in each group). The mortality rates of the fish were recorded every 12 h for 7 days to determine the mortality (Jia et al., 2020).

The bacterial loads in the crucian carp gut were determined. The crucian carp intestinal structure was harvested, weighed, and suspended in filter-sterilized PBS. The collected intestinal structure slurry was diluted. Then, 100 μL of each dilution was used to determine the bacterial loads.

A lipopolysaccharide (LPS) ELISA kit (Jiangsu Jingmei Biological Technology Co., Ltd., China) was used to measure the level of LPS in the crucian carp intestinal contents 24 h after phage treatment. The operation method complied with the product instructions.

### Histopathological Analysis

Histopathological analysis of the liver, spleen, kidney, gut, and gill was performed. Briefly, the crucian carp tissues were removed and placed in 4% formalin, stained with H&E, and analyzed by microscopy.

### Gut Microbiota Analysis

Totally, 24 crucian carps were randomly and equally divided into eight groups (Bg, Bge, Pg, Pge, BPg, BPge, Ng, and Nge). The fish in the Bg group were challenged i.p. with 2 × 10^8^ CFU of *A. hydrophila* 152 (per fish). The fish in the BPg group were challenged with *A. hydrophila* 152 (2 × 10^8^ CFU/fish) and injected i.p. with 2 × 10^9^ PFU of PZL-Ah152 after 1 h. The fish in the Pg group were injected i.p. with 2 × 10^9^ PFU of PZL-Ah152. The Ng group was not treated. The intestinal contents of each fish were collected at 24 h post-infection. The procedures for Bge, Pge, BPge, and Nge were the same as those for the previous four groups, except that the intestinal epithelial mucus was collected (Supplementary Table 1).

For each intestinal content and intestinal epithelial mucus sample, total genomic DNA was extracted by using a QIAamp DNA Stool Mini Kit (Qiagen, West Sussex, United Kingdom). Sequencing libraries were constructed by PCR amplification of the V3 + V4 regions of the 16S rRNA gene using the primers 341F (5′-CCTAYGGGRBGCASCAG-3′) and 806R (5′-GGACTACNNGGGTATCTAAT-3′) (Drengenes et al., 2021). The amplicons were purified using a Qiagen Gel Extraction Kit (Qiagen, Germany). Sequencing libraries were generated using the TruSeq^@^ DNA PCR-Free Sample Preparation Kit (Illumina, United States), and then, index codes were added. Library quality was assessed using a Qubit@ 2.0 fluorometer (Thermo Scientific) and an Agilent Bioanalyzer 2100 system.

Trimmomatic and PEAR were used to screen the FASTQ data and to obtain high-quality sequences. Raw tag quality filtering was performed under specific filtering conditions to obtain high-quality clean tags using QIIME (V1.7.0 3) (Caporaso et al., 2010; Bokulich et al., 2013). Sequence analyses were performed using UPARSE (UPARSE v7.0.1001 6) (Edgar et al., 2011). Sequence similarities (minimum identity of 97%) were assigned to the same OTUs (Febvre et al., 2019). Other subsequent biological analyses were conducted based on the results of the OTU cluster analysis.

### Statistical Analysis

All statistical analyses were performed using the SPSS (version 25). Standard deviations were calculated in all experiments. Redundancy analysis (RDA) was used to determine the correlation between intestinal cytokines and the composition of the intestinal microbiome. R software (version 2.15.3) was used for data analysis.

## Results

### Purification and Biological Characteristics of Phage

**FIGURE 1:**
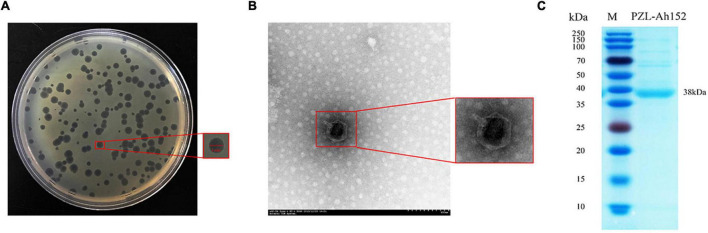
Phage morphology and structural protein detection. **(A)** Bacteriophage PZL-Ah152 spotted onto *A. hydrophila*-152 culture on LB agar. Each single plaque was ≈4 mm in diameter. **(B)** TEM image showing the icosahedral head and sheathed tail tube with the tail fiber of PZL-Ah152. Bar = 100 nm. **(C)** SDS–polyacrylamide gel (12%) electrophoresis of PZL-Ah152 structural proteins. M: molecular mass marker. Lane 1, PZL-Ah152 proteins. The most abundant structural protein was about 38 kDa.

The phage PZL-Ah152 was isolated from sewage systems in Changchun by plaque purification. PZL-Ah152 formed plaques after incubation for 12 h (3–4 mm diameter, Figure 1A). TEM images showed that PZL-Ah152 had a capsid diameter of 83 ± 1 nm and a tail length of 8 ± 1 nm. The morphological characteristics of PZL-Ah152 showed that it belonged to the *Podoviridae* family (Figure 1B). The structural protein of the most prominent protein band from phage capsids corresponded to the major capsid protein. The size indicated in the gel (38 kDa) (Figure 1C) was approximately the same as the ORF-01 protein (39.9 kDa) (Supplementary Table 2), as a potential major capsid protein.

**FIGURE 2:**
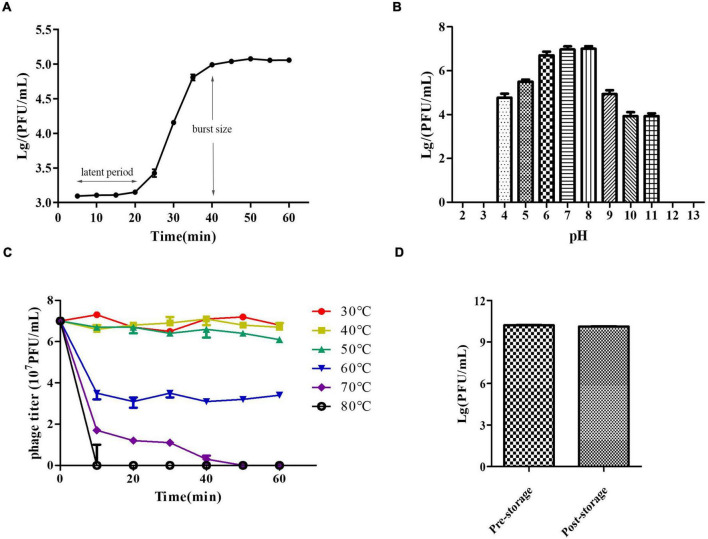
Growth characteristics and stability tests of PZL-Ah152. **(A)** One-step growth curve of PZL-Ah152. **(B)** pH stability: PZL-Ah152 were incubated under different pH values. **(C)** Thermal stability: phage particles were incubated at different temperatures as indicated. **(D)** Long-term storage stability: the titer of pre-storage and post-storage (for 1 year) of PZL-Ah152.

We determined the host range of PZL-Ah152 against a group of 40 *Aeromonas* spp. strains. Apart from *A. hydrophila* 152, PZL-Ah152 could lyse nine other *Aeromonas* strains, including eight strains of *A. hydrophila* (Ah-TPS, Ah-119, Ah-69, Ah-150, Ah-103, Ah-142, Ah-107, and Ah-87) and one strain of *Aeromonas veronii* AV-6 (Table 1). The results of the one-step growth curve showed that phage PZL-Ah152 expressed a short latent period of less than 20 min. The rise period was about 20 min. In addition, the burst size of phage PZL-Ah152 was approximately 91 PFUs per infected cell on strain *A. hydrophila* 152 (Figure 2A). PZL-Ah152 was stable at pH values in the range of 4-11 and acted more effectively at pH 6-8 (Figure 2B). The phage retained full activity at 30°C, 40°C, and 50°C but retained half of the activity at 60°C for the full time of exposure, and after 50 min of exposure to 70°C, the activity to kill bacteria disappeared. It was completely inactivated at 80°C after 10 min (Figure 2C). Furthermore, the titer of PZL-Ah152 remained almost constant after 1 year of storage at 4°C (Figure 2D).

### Genomic Characterization and Phylogenetic Analysis of PZL-Ah152

**FIGURE 3:**
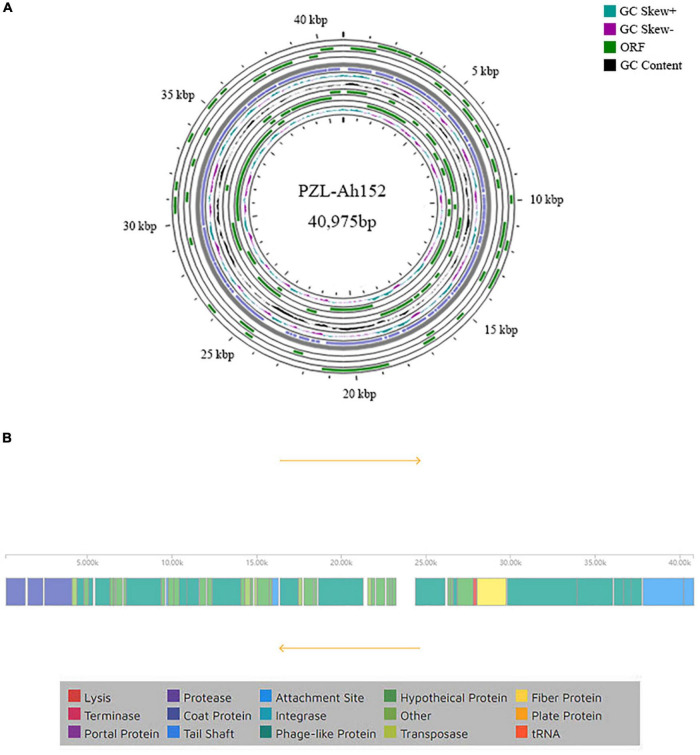
Genome characteristics of PZL-Ah152. **(A)** Circular representation of PZL-Ah152 genome. **(B)** Genetic and physical organization of the PZL-Ah152 genome.

To examine PZL-Ah152 at the genetic level, we obtained and analyzed the complete genome sequence of PZL-Ah152 and deposited it in GenBank (MW671054). The complete genome of PZL-Ah152 was 40,975 bp. The composition of the phage was 23.50% A, 24.76% T, 26.85% C, and 24.90% G (Figure 3A). The phage genome contained 54 putative ORFs (Supplementary Table 2). Among all 54 ORFs in PZL-Ah152, 52 ORFs (96.3%) had putative functions, and the sizes of the proteins encoded by the 52 ORFs ranged from 4.55 kDa (ORF15) to 149.96 kDa (ORF48).

BLASTp analysis showed that 23 proteins (42.6%) were annotated predictive functions, with the remaining 31 (53.7%) putative gene products as unknown functions. The 23 proteins according to their functions were divided into four categories: metabolism and replication of nucleic acids (ORFs 08, 18, 19, 22, 30, 31, and 34), host lysis (ORFs 17 and 46), structure/morphogenesis (ORFs 01, 02, 03, 27, 47, 48, 49, 50, 51, 53, and 54), and DNA packaging/maturation (ORF 44). The analysis did not identify any toxic genes in the PZL-Ah152 genome (Figure 3B).

**FIGURE 4:**
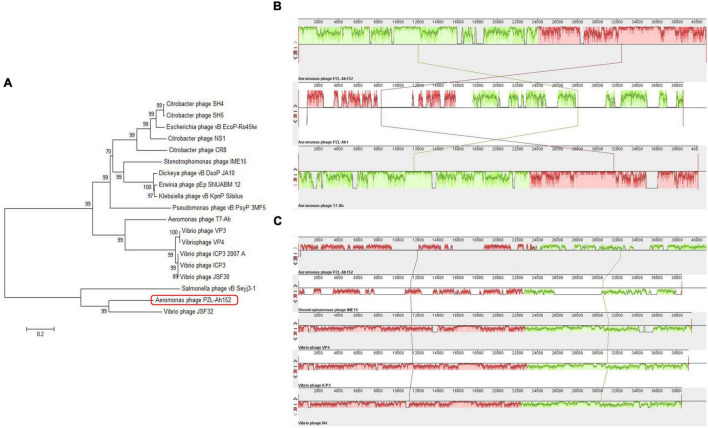
Evolutionary characteristics of PZL-Ah152. **(A)** Phylogenetic tree analysis of PZL-Ah152. **(B)** Multiple genome alignments among PZL-Ah152 and other homologous phages. Similarity is indicated by the height of the bars, complying with the average level of conservation in that region of the genome sequence. The homologous phages are marked in the picture. **(C)** Collinearity analysis of phage PZL-Ah152 with other phages.

The PZL-Ah152 genome was similar to the *Aeromonas* phage T7-Ah (95.76%), *Klebsiella* phage vB_KpnP_Sibilus (79.03%), *Dickeya* phage vB_DsoP_JA10 (78.85%), *Erwinia* phage pEp_SNUABM_12 (74.81%), *Vibrio* phage JSF30 (71.82%), *Vibrio* phage VP4 (71.41%), and *Vibrio* phage VP3 (71.35%) (Figures 4A–C), but the phage PZL-Ah152 genome also had its own unique sequences compared to those phages, such as the sequences from 6,529-6,645 bp, 7,132-7,290 bp, 9,361-9,570 bp, 15,917-16,279 bp, 17,798-18,361 bp, 18,405-18,563 bp, 22,670-22,861 bp, and 28,018-29,739 bp. This finding suggested that PZL-Ah152 is a new phage strain.

### Bacteriolytic Activity *in vitro*

**FIGURE 5:**
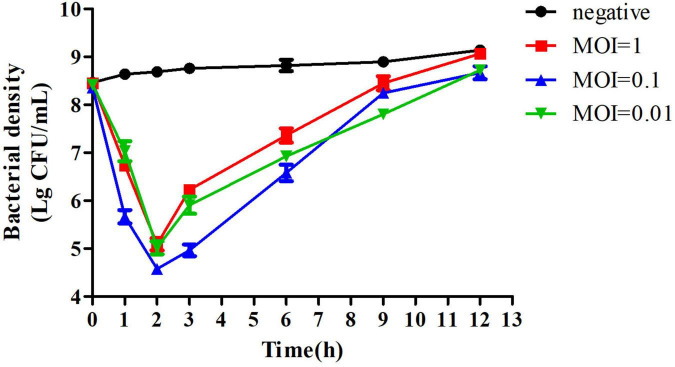
Bactericidal activity of PZL-Ah152 at different multiplicities of infection *in vitro*. The *A. hydrophila*-152 strain was lysed by phage PZL-Ah152 in LB medium at 37°C. CFU counts of the uninfected control culture and the parallel cultures that were infected with the phage at different MOI were measured over time. No phage was added in the negative control. The values represent the means and SD (*n* = 3).

To evaluate the activity of PZL-Ah152 against *A. hydrophila* 152, we performed a time killing assay. The results showed that when MOI = 0.1, the presence of phages after 2 h of co-incubation reduced *A. hydrophila* 152 counts by 4.2 log units. When MOI = 1 or MOI = 0.01, *A. hydrophila* 152 counts decreased by 3.95 and 3.67 log units after 2 h, respectively (Figure 5).

### PZL-Ah152 Improved the Survival Rate of Crucian Carps

**FIGURE 6:**
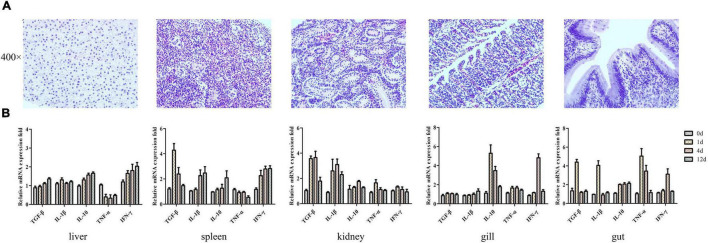
Transcriptional analysis of histopathology and immune-related genes (TGF-β, IL-1β, IL-10, TNF-α, and IFN-γ) in crucian carps upon exposure to PZL-Ah152 for 12 days. Experiments were conducted as independent duplicate experiments for control and phage exposure. Tissues were pooled (*n* = 3) at each time point for each replicate group. **(A)** The safety of using PZL-Ah152 as a potential therapeutic was examined by exposing crucian carps to PZL-Ah152 for 12 consecutive days. The tissues of crucian carp were extracted at 12 days and stained with hematoxylin and eosin. **(B)** Relative mRNA expression fold change for a particular candidate gene of PZL-Ah152 exposed fish at day 1 = 1.

The safety of using PZL-Ah152 as a potential therapeutic was examined by exposing crucian carps to PZL-Ah152 for 12 consecutive days. In the safety test, the survival rate of crucian carps reached 100%. In addition, the tissues collected from immunized crucian carps treated with phage showed no pathological changes, as shown in Figure 6A. The liver, spleen, kidney, gut, and gill tissues were surgically removed. The mRNA expression levels of TGF-β, IL-1β, TNF-α, IFN-γ, and IL-10 were quantified with qPCR. Compared with the control group, the transcription level of IL-10 mRNA in the liver and spleen began to increase on the first day, while the expression level of TNF-α decreased (Figure 6B). In the intestinal structure, the transcription levels of the five cytokines began to increase on the first day, and the mRNA expression level decreased to a non-significant level on the 12^th^ day.

**FIGURE 7:**
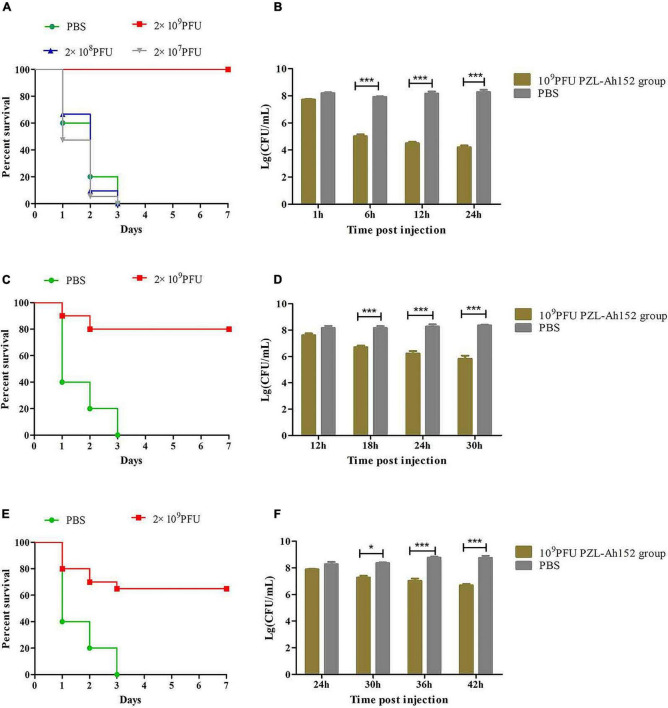
PZL-Ah152 therapeutic study. The fish were intraperitoneally injected with 2 × minimum lethal doses (MLD) (2 × 10^8^CFU/fish) of *A. hydrophila*-152. **(A,C,E)** Survival rate of different groups. One hours later, 10^7^, 10^8^, and 10^9^ PFU of PZL-Ah152 were introduced intraperitoneal **(A)**. After injection of *A. hydrophila*-152 (2 × 10^8^CFU/fish) 12 h later **(C)**, 24 h later **(E)**, 2 × 10^9^ PFU phage of PZL-Ah152 were introduced intraperitoneal. Control fish were administrated with PBS under the identical conditions. **(B,D,F)** Colony counts of bacteria changed in the gut. After injection of *A. hydrophila*-152 (2 × 10^8^CFU/fish) 1 h later **(B)**, 12 h later **(D)**, 24 h later **(F)**. Colony counts of bacteria changed in the gut at regular intervals (*n* = 6 in each group). Control fish were administrated with PBS under the identical conditions. The means and standard deviations are represented as points with error bars. *p < 0.05, ***p < 0.001.

The crucian carps were injected intraperitoneally with 2 × MLD (2 × 10^8^ CFU/fish) of *A. hydrophila* 152, and all of them died within 3 days. We administered intraperitoneal injections of different doses of the phage PZL-Ah152 1 h after challenging with *A. hydrophila* 152. Compared with PBS, bacteriophage (2 × 10^9^ PFU/fish) treatment significantly improved the survival rate (Figure 7A). After 1 h of *A. hydrophila* 152 (2 × 10^8^ CFU/fish) infection, the bacterial load reached > 10^7^ CFU/ml in the gut (Figure 7B). At the same time, crucian carps were administered with the phage PZL-Ah152 (1 × 10^10^ PFU/ml, 200 μL/fish). The bacterial load decreased by 3.8 log units in the gut tissues after 24 h (Figure 7B). The test produced a 100% survival rate within the experimental period (Figure 7A). In contrast, the bacterial loads in the gut were approximately 8.2 log units 12 h after the crucian carps were infected with *A. hydrophila* 152 (Figure 7D). Treatment with bacteriophage PZL-Ah152 (1 × 10^10^ PFU/ml, 200μL/fish) reduced the gut *A. hydrophila* by 2.6 log units after 18 h (Figure 7D) and produced 80% survival over 2 days (Figure 7C). However, 24 h after the crucian carps were infected with *A. hydrophila* 152, the use of bacteriophage PZL-Ah152 reduced the *A. hydrophila* 2 log unit in the crucian carp gut (Figure 7F) and resulted in a 65% survival rate within 3 days (Figure 7E). The results indicated that crucian carps should be treated with phage as soon as possible after bacterial infection.

**FIGURE 8:**
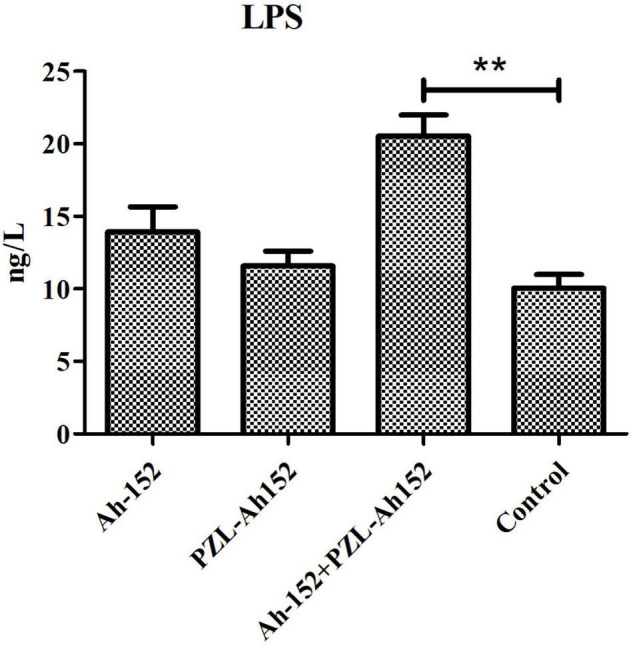
Determination of LPS content in intestinal contents of crucian carp. Ah-152: challenged i.p. with 2 × 10^8^ CFU of *A. hydrophila* 152 per fish. Ah-152+PZL-Ah152: challenged with *A. hydrophila* 152 (2 × 10^9^ CFU/ml, 100 μL/fish), 1 h later injected i.p. with 2 × 10^9^ of PZL-Ah152. PZL-Ah152: injected i.p. with 2 × 10^9^ PFU PZL-Ah152; Control: not treated. **p < 0.01.

By determining the LPS content in crucian carp intestines, we found that when the phage was applied, the LPS content increased along with the decrease in the *A. hydrophila* 152 level. Although the LPS content in the *A. hydrophila* 152-challenged group and the phage supplementation group increased, the increase was less significant than that in the control group (Figure 8).

**FIGURE 9:**
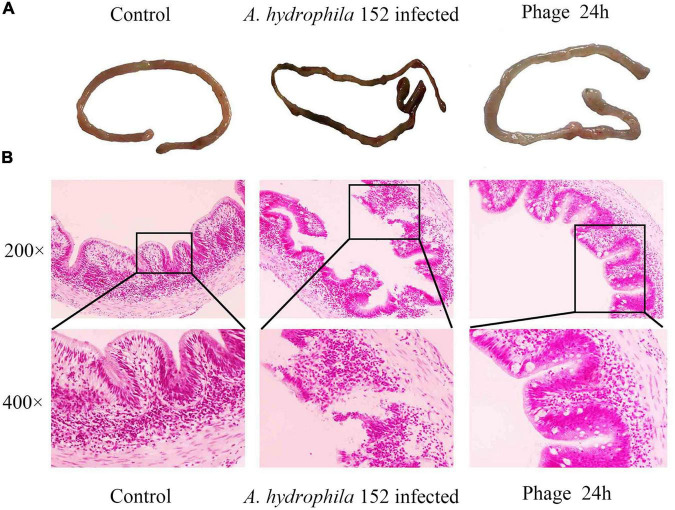
Gross pathology and histopathology of the gut tissue. **(A)** At 1 h post infection, the gut was removed from the fish that were treated with 2 × 10^9^ PZL-Ah152 or PBS. The gut of the healthy fish was used as controls. **(B)** The tissue samples were stained with haematoxylin and eosin.

Intestinal morphology is an important indicator of intestinal health. We evaluated the ameliorative effect of the phage PZL-Ah152 on intestinal pathological injury in crucian carps (Figure 9). Infection of crucian carps with *A. hydrophila* 152 caused damage to intestinal mucosa integrity and resulted in the loss of intestinal crypts, accompanied by inflammatory cell infiltration in the mucosa. The glands were not intact and showed obvious bleeding spots (Figure 9A). In contrast, after 24 h of treatment with the phage PZL-Ah152, the inflammation was gradually alleviated, and the intestinal villus injury progressively decreased, leading to intestinal crypts being observable. Accompanied by goblet cell production, the gut structure slowly returned to normal (Figure 9B).

### Effect of Bacteriophage on Intestinal Flora of Crucian Carps

**FIGURE 10:**
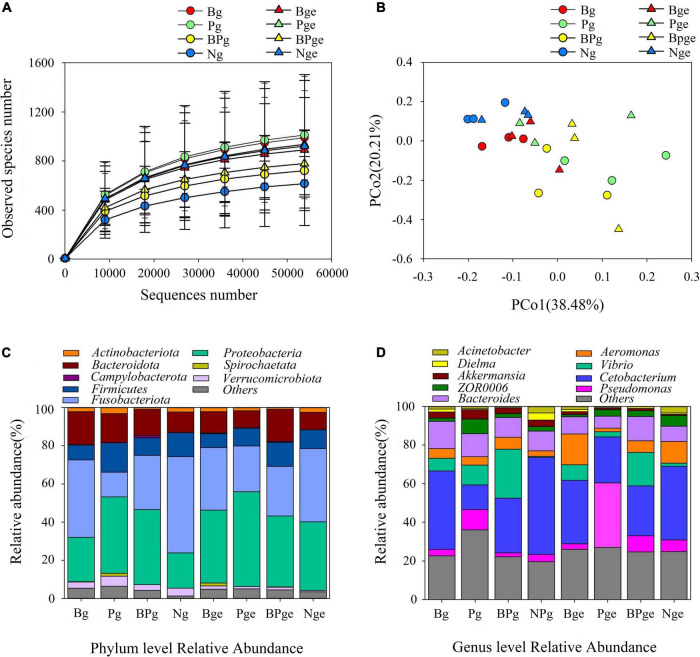
The effects of *A. hydrophila* challenge and phage therapy on the composition of the gut microbiota. **(A)** Rarefaction curves based on the observed species of bacteria. **(B)** PCoA of the bacteria community based on Bray-Curtis distance. **(C)** Bacterial taxonomic profiling at the phylum level. **(D)** Bacterial taxonomic profiling at the genus level.

The gut is the most important digestive organ of crucian carps. We analyzed the changes in the gut microbiota in crucian carps treated with phage therapy by selecting 1,538,144 effective tags. The plateauing of rarefaction curves in all the samples suggested that the sequencing depth was sufficient (Figure 10A). Moreover, the observed species of Ng in the intestinal contents was the smallest, but it was not significantly different from other treatments. The PCoA showed that there was a high similarity in the microbial community structures of the intestinal contents (Figure 10B). Upon comparing groups with and without phage treatment, we found that phage treatment had stronger effects on the intestinal microbial community of crucian carps. Compared with the Ng group, the *Proteobacteria* content increased significantly in each group (*P* < *0.05*), especially in the Pge group, which showed an increase of 21.5%. However, we discovered that the *Fusobacteriota* content decreased in either the intestinal contents (37.4%) or intestinal mucus (14.5%) in only the phage-treated groups (Figure 10C). In the intestinal contents and the intestinal epithelial mucus, the *Cetobacterium* abundance decreased by 10% and 5.3%, respectively, after challenged with *A. hydrophila* 152. In the Pg and Pge groups, the *Cetobacterium* abundance decreased by 37.3% and 14.3%, respectively, and in the BPg and BPge groups, the *Cetobacterium* abundance decreased by 22% and 12.3%, respectively. The *Vibrio* abundance increased by 5.8% and 6.4% in the intestinal contents and intestinal epithelial mucus, respectively, after *A. hydrophila* 152 was challenged; in the Pg and Pge groups, the *Vibrio* abundance increased by 9.4% and 1%, respectively, and in the BPg and BPge groups, the *Vibrio* abundance increased by 24.4% and 15.6%, respectively (Figure 10D). In addition, the crucian carps attacked by *A. hydrophila* 152 exhibited greater bacterial changes at the genus level than normal crucian carps. *Brevinema*, *Acidovorax*, and *Erythrobacter* in the intestinal epithelial mucus increased, as did the abundance of *Bacteroides*, in the intestinal contents (Supplementary Figure 1B). We also found that when the phage PZL-Ah152 was used to treat *A. hydrophila* 152 infection, the abundance of *Lactobacillus* in the intestinal epithelial mucus increased (Supplementary Figure 1B). The results indicated that *Aeromonas* in the Bge group was increased compared to that in the Nge group, while they were decreased in the Pge group. Thus, the addition of the bacteriophage resulted in a change in *Aeromonas* abundance in the epithelial mucus. However, this change in *Aeromonas* abundance was not observed in the microbial community of the intestinal contents (Figure 10D). Our study found that the intestinal flora abundance of crucian carps was also regulated by intestinal cytokines (Supplementary Figure 1A). The first and second axes accounted for 54.28% and 21.49% of the total variations, respectively. TNF-α was positively correlated with IL-1β, while TGF-β and IFN-γ were positively correlated with IL-10. Moreover, *Actinobacteriota* was closely related to TGF-β and IFN-γ transcription, *Firmicutes* was closely related to IL-10 expression, and *Proteobacteria* and *Bacteroidota* were closely related to TNF-α and IL-1β, respectively.

## Discussion

We isolated a new bacteriophage PZL-Ah152. Electron microscopy indicated that PZL-Ah152 had the morphological characteristics of the *Podoviridae* family. Stability is critical for the application of phages in clinical antimicrobial preparations. We found that PZL-Ah152 titers exhibited stability at pH 5–9 and temperatures < 50°C and could be maintained for long term at 4°C. Moreover, the genomic analysis did not identify any toxic genes in this phage. All these characteristics indicated that PZL-Ah152 could be a therapeutic agent against *A. hydrophila* infections.

We analyzed the genomic information of PZL-Ah152 in addition to its biological characteristics. The full genome of phage PZL-Ah152 was 40,975 bp, which was similar to that of the reported phages such as *Aeromonas* phage T7-Ah (GenBank number: MT740748.1) and PZL-Ah1 (GenBank number: MT681669.1). BLASTp results showed that the PZL-Ah152 genome was composed of four main groups: (1) genes involved in metabolism and replication of nucleic acids (ORFs 08, 18, 19, 22, 30, 31, and 34), (2) genes involved in host lysis (ORFs 17 and 46), (3) genes involved in structure/morphogenesis (ORFs 01, 02, 03, 27, 47, 48, 49, 50, 51, 53, and 54), and (4) genes involved in DNA packaging/maturation (ORF 44). When a virulent phage infected the bacterium, the phage needs to lyse the bacterium from the inside to release its progeny. This process required proteins of the phage with bacterial lysis-related functions. The phage lysis system is composed of lysin and holin proteins, while some phage lysis systems have only lysin-containing signal peptides. The phage PZL-Ah152 genome encoded both lysin (ORF 17) and holin (ORF 46). Holin and lysin genes are commonly adjacent to phage genomes. We found that these genes were split apart in phage PZL-Ah152, as was found in another *A. hydrophila* phage, PZL-Ah1 (GenBank number: MT681669.1). Our team will conduct detailed research on phage lysins in the future to improve the understanding of phage lysis systems.

When the phage PZL-Ah152 was cocultured with *A. hydrophila* 152 at different MOIs (MOI = 1, 0.1, and 0.01), phage-resistant bacteria began to develop 2 h after the start of the experiment (Figure 5). Bacteriophages with high titers may exert strong selective pressure on host bacteria, leading to the development of bacteriophage tolerance (Henein, 2013; Duerkop et al., 2016). We evaluated the therapeutic effect of the phage PZL-Ah152 in crucian carps. In this study, the survival rate of crucian carps reached 100% after 1 h of bacteriophage (2 × 10^9^ PFU/fish) treatment during a 7-day period. We found that the abundance of *A. hydrophila* in the gut was reduced by 2 log units using phage therapy after infection 24 h. This decrease seemed less significant than the phage-treated group at 12 h post-infection. The results showed that PZL-Ah152 could effectively reduce the number of *A. hydrophila* in the gut tract of crucian carps and improve the survival rate of crucian carps. However, when phage treatment was performed 12 or 24 h post-infection, the crucian carp survival rate decreased drastically, suggesting that bacteriophage treatment should be applied as early as possible. In this study, compared to crucian carps infected with *A. hydrophila* 152, the crucian carps in the bacteriophage treatment group had better intestinal morphology. Monsur found that a bacteriophage was as capable as tetracycline in alleviating diarrhea in patients without any apparent toxic effect (Monsur et al., 1970).

The safety of PZL-Ah152 as a potential therapeutic agent was confirmed by continuous injection of PZL-Ah152 into crucian carps for 12 days. In fact, bacteriophages can be used as growth promoters, which indicated the phage had good safety (Gebru et al., 2010). It was reported that bacteriophages were modulators of immune responses. We examined changes in TGF-β, IL-1β, IL-10, TNF-α, and IFN-γ levels in different tissues over a 12-day period. The liver and spleen were considered major organs involved in phage filtration and clearance. In these organs, phage titers are usually the highest compared to all the organs (Dabrowska et al., 2004; Chadha et al., 2017; Naghizadeh et al., 2019; Otero et al., 2019; Rouse et al., 2020; Prazak et al., 2021). In this study, the level of IL-10 gradually increased in the liver, spleen, and gut over time. Recent studies have shown that phages can induce IL-10 production in monocytes (Van Belleghem et al., 2017). This phenomenon, known as an anti-inflammatory effect, is thought to protect the liver from damage (Gorski et al., 2018; Van Belleghem et al., 2019). We also found that the IFN-γ levels increased after phage injection, and IFN-γ levels in the gills and gut returned to normal on day 12. One phage, named phage 536_P1, could directly promote the production of IFN-γ, IL-12, and chemokines in mouse lungs, even without a host bacterial infection (Dufour et al., 2019). In fact, experimental evidence in humans had indicated that the *Lactobacillus* and *E. coli* bacteriophages can induce IFN-γ production in a microbially dependent manner through TLR-9 in the gut, and thus, the induction triggers phage-specific immune responses and bacterial-specific immunity (Podlacha et al., 2021). In our research, the low level of immune response induced by PZL-Ah152 in crucian carps ensured the safety of phage adaptation in phage therapy. Nevertheless, the interaction between different phage administration modalities and bacterial infection needed to be deeply examined. As foreign bodies, bacteriophages can induce innate and adaptive immune responses, in addition to the direct immune responses to the phage (Krut and Bekeredjian-Ding, 2018). The endotoxin (LPS) produced after phage bacterial elimination can also activate the innate immune response (Ogikubo et al., 2004; Sahoo et al., 2017). Our results showed that LPS levels in the crucian carp intestinal contents increased after phage treatment (*P* < *0.01*) (Figure 8). We speculated that this increase could be attributed to the phage-mediated lysis of the bacteria. Low-dose LPS can enhance the non-specific immunity of the body by activating the complement replacement pathway, phagocytic activity of macrophages, and proliferation of B and T lymphocytes (Nya and Austin, 2010). Studies have shown that the coordinated action of bacteriophages and the innate immune system was crucial to the clearance of *Pseudomonas aeruginosa* (Roach et al., 2017). In addition, a study showed that the addition of bacteriophage to the diet promoted the expression of TLR2, TLR4, and TLR9 mRNA in the jejunum mucosa of pigs, indicating that the bacteriophage activated the immune system by regulating TLR response (Zeng et al., 2021).

The gut microbiota are a highly complex ecosystem. Studies have shown that the balance of the intestinal microflora plays a key role in regulating the immune system of the host (Shi et al., 2017). A recent study found that phage predation not only reduced the relative abundance of target bacteria in the gut but also led to changes in untargeted bacteria through bacterial interactions, resulting in changes in certain bacteria (Cheng et al., 2017). These phage-mediated changes in the microbiome further regulated the metabolic activity of the gut microbiota (Belizario and Faintuch, 2018). In our study, we evaluated whether the treatment of *A. hydrophila* with a bacteriophage affects the gut microbiota balance of crucian carps, and our results showed that the bacteriophage preparation maintains the natural richness and diversity of the gut commensal flora in the fish. We found that the dominant bacterial taxa in the gut of crucian carps were *Bacteroidota*, *Firmicutes*, *Fusobacteriota*, and *Proteobacteria*. Our study showed that there were slight changes in the abundance of *Fusobacteriota* and *Proteobacteria* in the Pg, BPg, Pge, and BPge groups, but compared with the control group, the differences were not significant (*P* > *0.05*), where the abundance of *Bacteroidota* and *Firmicutes* did not change. Studies have shown that *Firmicutes* can improve energy efficiency in diets (Ley et al., 2006). The ratio of *Firmicutes* to *Bacteroidota* was generally positively correlated with body weight gain (Bervoets et al., 2013). *A. hydrophila* is a common pathogen in fish and mainly exists in the lower part of the gut. When crucian carps were injected with 2 x MLD of *A. hydrophila* 152, the abundance of *Aeromonas* in the intestinal contents and intestinal epithelial mucus increased, leading to the death of all crucian carps in the experiment. After treatment with phage PZL-Ah152, the abundance of *A. hydrophila* in the intestinal epithelial mucus of crucian carps significantly decreased. We speculated that *A. hydrophila* was more likely to adhere to the intestinal mucosa of crucian carps. However, the host bacteria were not completely eliminated and instead reached a state of coexistence with the phage. We also found that changes in the abundance of *Aeromonas* changed the abundance of other bacteria in the gut. We noticed that after phage treatment, the abundance of *Vibrio* in the gut increased. We thought that this increase was caused by the decrease in *Aeromonas* abundance in the gut, leading to increased reproduction of *Vibrio* bacteria that originally existed in the intestinal mucosa. However, the precise reasons need to be further determined.

## Conclusion

In this study, we isolated phage PZL-Ah152 which was proven safe and therapeutically effective against the enteritis of crucian carps caused by *A. hydrophila*. The application of bacteriophages indeed brought changes in the gut microbiota of crucian carps without disrupting the gut microbiota balance. All these data suggested that phage therapy should be regarded as feasible for treating *A. hydrophila* infection.

## Data Availability Statement

The data that support the findings of this study are available from the corresponding authors.

## Ethics Statement

The animal study was reviewed and approved by Animal Welfare and Research Ethics Committee at Jilin Agriculture University.

## Author Contributions

CF, KJ, SH, AA, LeZ, XS, AQ, WS, and DZ conceived to the study. CF, KJ, TC, SC, HY, Laz, SL, ZZ, TL, and YQ performed the experiments. All authors contributed to manuscript revision, read, and approved the submitted version.

## Conflict of Interest

The authors declare that the research was conducted in the absence of any commercial or financial relationships that could be construed as a potential conflict of interest.

## Publisher’s Note

All claims expressed in this article are solely those of the authors and do not necessarily represent those of their affiliated organizations, or those of the publisher, the editors and the reviewers. Any product that may be evaluated in this article, or claim that may be made by its manufacturer, is not guaranteed or endorsed by the publisher.

## Supplementary Material

Supplementary Figure 1 The effects of *A. hydrophila* challenge and phage therapy on the composition of the gut microbiota. **(A)** Redundancy analysis (RDA) revealed the relationship between inflammatory factors and Intestinal flora in different groups. **(B)** Heatmapping shows that the abundance of intestinal bacteria at the genus level was significantly altered by treatment with phage PZL-Ah152 in challenged and normal fish.

## References

[B1] Akmal M. Rahimi-Midani A. Hafeez-ur-Rehman M. Hussain A. Choi T. (2020). Isolation, characterization, and application of a bacteriophage infecting the fish pathogen *Aeromonas hydrophila*. *Pathogens* 9:215. 10.3390/pathogens9030215 32183136 PMC7157608

[B2] Belizario J. E. Faintuch J. (2018). Microbiome and gut dysbiosis. *Exp. Suppl.* 109 459–476. 10.1007/978-3-319-74932-7_13 30535609

[B3] Bervoets L. Van Hoorenbeeck K. Kortleven I. Van Noten C. Hens N. Vael C. (2013). Differences in gut microbiota composition between obese and lean children: a cross-sectional study. *Gut Pathog.* 5:10. 10.1186/1757-4749-5-10 23631345 PMC3658928

[B4] Bokulich N. A. Subramanian S. Faith J. J. Gevers D. Gordon J. I. Knight R. (2013). Quality-filtering vastly improves diversity estimates from illumina amplicon sequencing. *Nat. Methods* 10 11–57. 10.1038/NMETH.2276 23202435 PMC3531572

[B5] Caporaso J. G. Kuczynski J. Stombaugh J. Bittinger K. Bushman F. D. Costello E. K. (2010). QIIME allows analysis of high-throughput community sequencing data. *Nat. Methods* 7 335–336. 10.1038/nmeth.f.303 20383131 PMC3156573

[B6] Chadha P. Katare O. P. Chhibber S. (2017). Liposome loaded phage cocktail: enhanced therapeutic potential in resolving *Klebsiella pneumoniae* mediated burn wound infections. *Burns* 43 1532–1543. 10.1016/j.burns.2017.03.029 28502784

[B7] Cheng M. Liang J. Zhang Y. Hu L. Gong P. Cai R. (2017). The bacteriophage ef-p29 efficiently protects against lethal vancomycin-resistant enterococcus faecalis and alleviates gut microbiota imbalance in a murine bacteremia model. *Front. Microbiol.* 8:837. 10.3389/fmicb.2017.00837 28536572 PMC5423268

[B8] Citterio B. Francesca B. (2015). *Aeromonas hydrophila* virulence. *Virulence* 6 417–418. 10.1080/21505594.2015.1058479 26055576 PMC4601520

[B9] Dabrowska K. Opolski A. Wietrzyk J. Switala-Jelen K. Godlewska J. Boratynski J. (2004). Anticancer activity of bacteriophage t4 and its mutant hap1 in mouse experimental tumour models. *Anticancer Res.* 24 3991–3995. 15736444

[B10] Danis-Wlodarczyk K. Dabrowska K. Abedon S. T. (2021). Phage therapy: the pharmacology of antibacterial viruses. *Curr. Issues Mol. Biol.* 40 81–163. 10.21775/cimb.040.081 32503951

[B11] Drengenes C. Eagan T. M. L. Haaland I. Wiker H. G. Nielsen R. (2021). Exploring protocol bias in airway microbiome studies: one versus two PCR steps and 16s rRNA gene region v3 v4 versus v4. *BMC Genomics* 22:3. 10.1186/s12864-020-07252-z 33397283 PMC7784388

[B12] Duerkop B. A. Huo W. Bhardwaj P. Palmer K. L. Hooper L. V. (2016). Molecular basis for lytic bacteriophage resistance in enterococci. *mBio* 7 1304–1320. 10.1128/mBio.01304-16 27578757 PMC4999554

[B13] Dufour N. Delattre R. Chevallereau A. Ricard J. Debarbieux L. (2019). Phage therapy of pneumonia is not associated with an overstimulation of the inflammatory response compared to antibiotic treatment in mice. *Antimicrob. Agents Chemother.* 63:e00379-19. 10.1128/AAC.00379-19 31182526 PMC6658787

[B14] Duplessis C. A. Biswas B. (2020). A review of topical phage therapy for chronically infected wounds and preparations for a randomized adaptive clinical trial evaluating topical phage therapy in chronically infected diabetic foot ulcers. *Antibiotics* 9:377. 10.3390/antibiotics9070377 32635429 PMC7400337

[B15] Easwaran M. Dananjaya S. H. S. Park S. C. Lee J. Shin H. De Zoysa M. (2017). Characterization of bacteriophage pah-1 and its protective effects on experimental infection of *Aeromonas hydrophila* in zebrafish (*Danio rerio*). *J. Fish Dis.* 40 841–846. 10.1111/jfd.12536 27454188

[B16] Edgar R. C. Haas B. J. Clemente J. C. Quince C. Knight R. (2011). Uchime improves sensitivity and speed of chimera detection. *Bioinformatics* 27 2194–2200. 10.1093/bioinformatics/btr381 21700674 PMC3150044

[B17] Febvre H. P. Rao S. Gindin M. Goodwin N. D. M. Finer E. Vivanco J. S. (2019). Phage study: effects of supplemental bacteriophage intake on inflammation and gut microbiota in healthy adults. *Nutrients* 11:666. 10.3390/nu11030666 30897686 PMC6471193

[B18] Gebru E. Lee J. S. Son J. C. Yang S. Y. Shin S. A. Kim B. (2010). Effect of probiotic-, bacteriophage-, or organic acid-supplemented feeds or fermented soybean meal on the growth performance, acute-phase response, and bacterial shedding of grower pigs challenged with *Salmonella enterica* serotype Typhimurium. *J. Anim. Sci.* 88 3880–3886. 10.2527/jas.2010-2939 20729283

[B19] Gong P. Cheng M. Li X. Jiang H. Yu C. Kahaer N. (2016). Characterization of *Enterococcus faecium* bacteriophage ime-efm5 and its endolysin lysefm5. *Virology* 492 11–20. 10.1016/j.virol.2016.02.006 26896930

[B20] Gorski A. Jonczyk-Matysiak E. Lusiak-Szelachowska M. Weber-Dabrowska B. Miedzybrodzki R. Borysowski J. (2018). Therapeutic potential of phages in autoimmune liver diseases. *Clin. Exp. Immunol.* 192 1–6. 10.1111/cei.13092 29266228 PMC5842411

[B21] Gu J. Xu W. Lei L. Huang J. Feng X. Sun C. (2011). Lysgh15, a novel bacteriophage lysin, protects a murine bacteremia model efficiently against lethal methicillin-resistant *Staphylococcus aureus* infection. *J. Clin. Microbiol.* 49 111–117. 10.1128/JCM.01144-10 21048011 PMC3020447

[B22] Henein A. (2013). What are the limitations on the wider therapeutic use of phage? *Bacteriophage* 3:e24872. 10.4161/bact.24872 24228220 PMC3821673

[B23] Hsu B. B. Gibson T. E. Yeliseyev V. Liu Q. Lyon L. Bry L. (2019). Dynamic modulation of the gut microbiota and metabolome by bacteriophages in a mouse model. *Cell Host Microbe* 25 803–814.e5. 10.1016/j.chom.2019.05.001 31175044 PMC6579560

[B24] Hyman P. (2019). Phages for phage therapy: isolation, characterization, and host range breadth. *Pharmaceuticals* 12:35. 10.3390/ph12010035 30862020 PMC6469166

[B25] Jia K. Yang N. Zhang X. Cai R. Zhang Y. Tian J. (2020). Genomic, morphological and functional characterization of virulent bacteriophage ime-jl8 targeting *Citrobacter freundii*. *Front. Microbiol.* 11:585261. 10.3389/fmicb.2020.585261 33329451 PMC7717962

[B26] Jikia D. Chkhaidze N. Imedashvili E. Mgaloblishvili I. Tsitlanadze G. Katsarava R. (2005). The use of a novel biodegradable preparation capable of the sustained release of bacteriophages and ciprofloxacin, in the complex treatment of multidrug-resistant *Staphylococcus aureus*-infected local radiation injuries caused by exposure to sr90. [Case Reports; Journal Article; Research Support, Non-U.S. Gov’t]. *Clin. Exp. Dermatol.* 30 23–26. 10.1111/j.1365-2230.2004.01600.x 15663496

[B27] Jun J. W. Kim J. H. Shin S. P. Han J. E. Chai J. Y. Park S. C. (2013). Protective effects of the aeromonas phages pah1-c and pah6-c against mass mortality of the cyprinid loach (*Misgurnus anguillicaudatus*) caused by *Aeromonas hydrophila*. *Aquaculture* 416-417 289–295. 10.1016/j.aquaculture.2013.09.045

[B28] Kostic A. D. Howitt M. R. Garrett W. S. (2013). Exploring host-microbiota interactions in animal models and humans. *Genes Dev.* 27 701–718. 10.1101/gad.212522.112 23592793 PMC3639412

[B29] Kropinski A. M. (2017). *Practical Advice on the One-Step Growth Curve*, Vol. 1681. New York, NY: Springer, 41–47. 10.1007/978-1-4939-7343-9_3 29134585

[B30] Krut O. Bekeredjian-Ding I. (2018). Contribution of the immune response to phage therapy. *J. Immunol.* 200 3037–3044. 10.4049/jimmunol.1701745 29685950

[B31] Ley R. E. Turnbaugh P. J. Klein S. Gordon J. I. (2006). Microbial ecology - human gut microbes associated with obesity. *Nature* 444 1022–1023. 10.1038/4441022a 17183309

[B32] Liu J. Gao S. Dong Y. Lu C. Liu Y. (2020). Isolation and characterization of bacteriophages against virulent *Aeromonas hydrophila*. *BMC Microbiol.* 20:141. 10.1186/s12866-020-01811-w 32487015 PMC7268745

[B33] Liu X. Sun W. Zhang Y. Zhou Y. Xu J. Gao X. (2020). Impact of *Aeromonas hydrophila* and infectious spleen and kidney necrosis virus infections on susceptibility and host immune response in Chinese perch (*Siniperca chuatsi*). *Fish Shellfish Immunol*. 105 117–125. 10.1016/j.fsi.2020.07.012 32653585

[B34] Monsur K. A. Rahman M. A. Huq F. Islam M. N. Northrup R. S. Hirschhorn N. (1970). Effect of massive doses of bacteriophage on excretion of vibrios, duration of diarrhoea and output of stools in acute cases of cholera. *Bull. World Health Organ.* 42 723–732. 4988693 PMC2427496

[B35] Naghizadeh M. Torshizi M. A. K. Rahimi S. Engberg R. M. Dalgaard T. S. (2019). Effect of serum anti-phage activity on colibacillosis control by repeated phage therapy in broilers. *Vet. Microbiol.* 234 61–71. 10.1016/j.vetmic.2019.05.018 31213273

[B36] Nya E. J. Austin B. (2010). Use of bacterial lipopolysaccharide (LPS) as an immunostimulant for the control of *Aeromonas hydrophila* infections in rainbow trout *Oncorhynchus mykiss* (Walbaum). *J. Appl. Microbiol.* 108 686–694. 10.1111/j.1365-2672.2009.04464.x 19674184

[B37] Ogikubo Y. Norimatsu M. Sasaki Y. Yasuda A. Saegusa J. Tamura Y. (2004). Effect of lipopolysaccharide (LPS) injection on the immune responses of LPS-sensitive mice. *J. Vet. Med. Sci*. 66 1189–1193. 10.1292/jvms.66.1189 15528847

[B38] Otero J. Garcia-Rodriguez A. Cano-Sarabia M. Maspoch D. Marcos R. Cortes P. (2019). Biodistribution of liposome-encapsulated bacteriophages and their transcytosis during oral phage therapy. *Front. Microbiol.* 10:689. 10.3389/fmicb.2019.00689 31019499 PMC6458305

[B39] Podlacha M. Grabowski L. Kosznik-Kawsnicka K. Zdrojewska K. Stasilojc M. Wegrzyn G. (2021). Interactions of bacteriophages with animal and human organisms-safety issues in the light of phage therapy. *Int. J. Mol. Sci.* 22:8937. 10.3390/ijms22168937 34445641 PMC8396182

[B40] Prazak J. Valente L. G. Iten M. Federer L. Grandgirard D. Soto S. (2021). Benefits of aerosolized phages for the treatment of pneumonia due to methicillin-resistant *Staphylococcus aureus*: an experimental study in rats. *J. Infect. Dis.* 225 1452–1459. 10.1093/infdis/jiab112 33668071 PMC9016458

[B41] Rawls J. F. Samuel B. S. Gordon J. I. (2004). Gnotobiotic zebrafish reveal evolutionarily conserved responses to the gut microbiota. *Proc. Natl. Acad. Sci. U.S.A.* 101 4596–4601. 10.1073/pnas.0400706101 15070763 PMC384792

[B42] Roach D. R. Leung C. Y. Henry M. Morello E. Singh D. Di Santo J. P. (2017). Synergy between the host immune system and bacteriophage is essential for successful phage therapy against an acute respiratory pathogen. *Cell Host Microbe* 22 38–47.e4. 10.1016/j.chom.2017.06.018 28704651

[B43] Rouse M. D. Stanbro J. Roman J. A. Lipinski M. A. Jacobs A. Biswas B. (2020). Impact of frequent administration of bacteriophage on therapeutic efficacy in an *A. baumannii* mouse wound infection model. *Front. Microbiol.* 11:414. 10.3389/fmicb.2020.00414 32256472 PMC7090133

[B44] Sahoo L. Parhi J. Debnath C. Prasad K. P. (2017). Effect of feeding lipopolysaccharide as an immunostimulant on immune response and immune gene expression of *Labeo bata*. *Vet. Immunol. Immunopathol.* 188 48–58. 10.1016/j.vetimm.2017.04.012 28615127

[B45] Shi N. Li N. Duan X. Niu H. (2017). Interaction between the gut microbiome and mucosal immune system. *Mil. Med. Res.* 4:14. 10.1186/s40779-017-0122-9 28465831 PMC5408367

[B46] Tsujimoto Y. Kanzawa Y. Seto H. Nakajima T. Ishimaru N. Waki T. (2019). Necrotizing fasciitis and sepsis caused by *Aeromonas hydrophila*. *Infez. Med*. 27 429–435. 31846994

[B47] Van Belleghem J. D. Clement F. Merabishvili M. Lavigne R. Vaneechoutte M. (2017). Pro- and anti-inflammatory responses of peripheral blood mononuclear cells induced by *Staphylococcus aureus* and *Pseudomonas aeruginosa* phages. *Sci. Rep.* 7:8004. 10.1038/s41598-017-08336-9 28808331 PMC5556114

[B48] Van Belleghem J. D. Dabrowska K. Vaneechoutte M. Barr J. J. Bollyky P. L. (2019). Interactions between bacteriophage, bacteria, and the mammalian immune system. *Viruses* 11:10. 10.3390/v11010010 30585199 PMC6356784

[B49] Yu H. Zhang L. Feng C. Chi T. Qi Y. Abbas Raza S. H. (2022). A phage cocktail in controlling phage resistance development in multidrug resistant *Aeromonas hydrophila* with great therapeutic potential. *Microb. Pathog*. 162:105374. 10.1016/j.micpath.2021.105374 34968644

[B50] Yun S. Jun J. W. Giri S. S. Kim H. J. Chi C. Kim S. G. (2019). Immunostimulation of *Cyprinus carpio* using phage lysate of *Aeromonas hydrophila*. *Fish Shellfish Immunol.* 86 680–687. 10.1016/j.fsi.2018.11.076 30513387

[B51] Zeng Y. Wang Z. Zou T. Chen J. Li G. Zheng L. (2021). Bacteriophage as an alternative to antibiotics promotes growth performance by regulating intestinal inflammation, intestinal barrier function and gut microbiota in weaned piglets. *Front. Vet. Sci.* 8:623899. 10.3389/fvets.2021.623899 33585620 PMC7874526

[B52] Zhu W. Zhou S. Chu W. (2020). Comparative proteomic analysis of sensitive and multi-drug resistant *Aeromonas hydrophila* isolated from diseased fish. *Microb. Pathog.* 139:103930. 10.1016/j.micpath.2019.103930 31846742

