# Supplementary material for: Lytic Bacteriophage PZL-Ah152 as Biocontrol Measures Against Lethal Aeromonas hydrophila Without Distorting Gut Microbiota
Source: Front Microbiol. 2022 Jul 12;13:898961. doi: 10.3389/fmicb.2022.898961 (PMC9315158; doi:10.3389/fmicb.2022.898961)
Supplement: Supplementary file 1 [file Data_Sheet_1.docx]

**Table S1. Grouping of intestinal microbiome analysis test**

| Sampling site | Group name | Methods |
| --- | --- | --- |
| Intestinal contents | Bg | Challenged with *A. hydrophila* 152 (2 × 10^8^ CFU/ fish) |
|  | BPg | Challenged with *A. hydrophila* 152 (2 × 10^8^ CFU/ fish) and injected with PZL-Ah152 (2×10^9^ PFU/fish) after 1 h |
|  | Pg | Injected with PZL-Ah152 (2×10^9^ PFU/fish) |
|  | Ng | Without any treatment |
| Intestinal epithelial mucus | Bge | Challenged with *A. hydrophila* 152 (2 × 10^8^ CFU/ fish) |
|  | BPge | Challenged with *A. hydrophila* 152 (2 × 10^8^ CFU/ fish) and injected with PZL-Ah152 (2×10^9^ PFU/fish) after 1 h |
|  | Pge | Injected with PZL-Ah152 (2×10^9^ PFU/fish) |
|  | Nge | Without any treatment |
