# Supplementary material for: Lytic Bacteriophage PZL-Ah152 as Biocontrol Measures Against Lethal Aeromonas hydrophila Without Distorting Gut Microbiota
Source: Front Microbiol. 2022 Jul 12;13:898961. doi: 10.3389/fmicb.2022.898961 (PMC9315158; doi:10.3389/fmicb.2022.898961)
Supplement: Supplementary file 2 [file Data_Sheet_2.docx]

**Table S2. General features of the putative ORFs from PZL-Ah152 with the best matches in the database**

| **ORFs** | **Start** | **End** | **Amino acid** | **Protein size (kDa)** | **pI** | **Annotated function** | **Best homolog** | **E**  **value** | **Identity (positives)** | **Query cover** | **Accession no.** |
| --- | --- | --- | --- | --- | --- | --- | --- | --- | --- | --- | --- |
| ORF-01 | 194 | 1321 | 375 | 39.93 | 6.26 | major/minor capsid protein | [Vibrio phage VP3] | 0 | 75 %（83 %） | 94 % | AFH14436.1 |
| ORF-02 | 1479 | 2372 | 297 | 32.02 | 4.5 | Capsid assembly protein | [Vibriophage VP4] | 1.00E-62 | 44 %（59 %） | 100 % | YP_249588.2 |
| ORF-03 | 2469 | 4085 | 538 | 59.32 | 4.78 | head-to-tail joining protein | [Vibrio phage JSF31] | 0 | 71 %（84 %） | 95 % | YP_249588.2 |
| ORF-04 | 4096 | 4383 | 95 | 10.11 | 9.69 | hypothetical protein | [Aeromonas phage T7-Ah] | 4e-56 | 98 %  (100 %) | 49 % | QOC54764.1 |
| ORF-05 | 4384 | 4779 | 131 | 15.45 | 6.09 | hypothetical protein | [Vibrio phage VP3] | 5e-09 | 35 %  (56 %) | 77 % | AFH14432.1 |
| ORF-06 | 4801 | 5052 | 83 | 8.54 | 9.17 | hypothetical protein | [Vibrio phage VP3] | 1e-18 | 55 %  (65 %） | 95 % | AFH14431.1 |
| ORF-07 | 5064 | 5318 | 84 | 9.5 | 4.34 | hypothetical protein | [Aeromonas phage T7-Ah] | 1e-51 | 98 %  (97 %) | 100 % | QOC54764.1 |
| ORF-08 | 5464 | 6333 | 289 | 33.08 | 5.15 | exonuclease | [Vibrio phage ICP3] | 2e-133 | 62 %（76 %） | 100 % | YP_004251272.1 |
| ORF-09 | 6330 | 6536 | 68 | 7.48 | 9.1 | hypothetical protein | [Aeromonas phage T7-Ah] | 4e-43 | 100 %  (100 %) | 100 % | QOC54764.1 |
| ORF-10 | 6529 | 6645 | 38 | 4.62 | 4.97 | hypothetical protein | [Aeromonas phage T7-Ah] | 4e-18 | 100 %  (100 %) | 100 % | QOC54764.1 |
| ORF-11 | 6693 | 7049 | 118 | 13.27 | 5.19 | hypothetical protein | [Vibrio phage VP3] | 1e-06 | 34 % (53 %) | 81 % | AFH14419.1 |
| ORF-12 | 7132 | 7290 | 52 | 5.88 | 8.8 |  |  |  |  |  |  |
| ORF-13 | 7300 | 9351 | 683 | 76.78 | 7.62 | hypothetical protein | [Vibrio phage Rostov-1] | 5e-164 | 79 %  (87 %) | 100 % | AVH85436.1 |
| ORF-14 | 9361 | 9570 | 69 | 7.9 | 9.15 |  |  |  |  |  |  |
| ORF-15 | 9655 | 9774 | 39 | 4.55 | 7.81 | hypothetical protein | [Citrobacter phage CF1 DK-2017] | 2e-05 | 54 %  (70 %) | 94 % | ARK07666.1 |
| ORF-16 | 9774 | 10115 | 113 | 12.48 | 9.13 | hypothetical protein | [Pseudomonas virus PBPA162] | 1e-65 | 82 %  (93 %) | 100 % | QDB70879.1 |
| ORF-17 | 10108 | 10422 | 104 | 11.86 | 6.51 | lysozyme | [Pseudomonas phage PFP1] | 1e-06 | 44 %  (59 %) | 59 % | AWY10467.1 |
| ORF-18 | 10419 | 10874 | 151 | 17.29 | 9.39 | endonuclease | [Vibrio phage ICP3] | 9e-90 | 84 %  (93 %) | 98 % | YP_004251274.1 |
| ORF-19 | 10874 | 11590 | 238 | 26.52 | 5.08 | ssDNA-binding protein | [Vibriophage VP4] | 6e-54 | 54 %  (67 %) | 79 % | YP_249584.1 |
| ORF-20 | 11630 | 12007 | 125 | 13.25 | 9.62 | hypothetical protein | [Sinorhizobium medicae] | 6e-06 | 37 %  (53 %) | 83 % | WP_153493865.1 |
| ORF-21 | 12077 | 12346 | 89 | 10.07 | 10.5 | hypothetical protein | [Aeromonas phage T7-Ah] | 7e-42 | 96 %  (97 %) | 100 % | QOC54764.1 |
| ORF-22 | 12375 | 14069 | 564 | 62.35 | 5.26 | DNA primase/helicase | [Vibrio phage N4] | 0 | 65 %  (81 %) | 98 % | YP_003347912.1 |
| ORF-23 | 14062 | 14286 | 74 | 8.52 | 6.05 | hypothetical protein | [Aeromonas phage T7-Ah] | 7e-42 | 91 %  (94 %) | 100 % | QOC54764.1 |
| ORF-24 | 14297 | 14620 | 107 | 12.42 | 9.56 | hypothetical protein | [Aeromonas phage T7-Ah] | 2e-73 | 98 %  (99 %) | 100 % | QOC54764.1 |
| ORF-25 | 14639 | 14779 | 46 | 5.88 | 10.4 | hypothetical protein | [Aeromonas phage T7-Ah] | 2e-23 | 100 %  (100 %) | 100 % | QOC54764.1 |
| ORF-26 | 14871 | 15035 | 54 | 6.26 | 4.67 | hypothetical protein | [Aeromonas phage T7-Ah] | 3e-31 | 98 %  (100 %) | 100 % | QOC54764.1 |
| ORF-27 | 15050 | 15727 | 225 | 26.14 | 6.06 | virion structural protein | [Pseudomonas phage phi15] | 1e-18 | 31 %（50 %） | 96 % | APD19628.1 |
| ORF-28 | 15752 | 15892 | 46 | 5.08 | 8.29 | hypothetical protein | [Aeromonas phage T7-Ah] | 3e-24 | 96 %  (100 %) | 100 % | QOC54764.1 |
| ORF-29 | 15917 | 16279 | 120 | 13.47 | 5.46 | putative nucleotide | [Pseudomonas phage PaMx25] | 1e-44 | 62 %  (75 %) | 100 % | YP_009603607.1 |
| ORF-30 | 16385 | 17455 | 356 | 39.72 | 6.09 | putative DNA ligase | [Klebsiella phage vB_KpnP_KpV763] | 2e-82 | 55 %（68 %） | 99 % | AOT28139.1 |
| ORF-31 | 17468 | 17680 | 70 | 8.15 | 9.78 | DNA ligase | [Vibrio phage JSF36] | 4e-30 | 52 %  (66 %) | 62 % | ASV43148.1 |
| ORF-32 | 17798 | 18361 | 187 | 21.26 | 5.8 | hypothetical protein | [Fusobacteriia bacterium 4572_132] | 2e-36 | 38 %  (58 %) | 96 % | OQY10304.1 |
| ORF-33 | 18405 | 18563 | 52 | 6.09 | 5 | hypothetical protein | [Escherichia coli] | 4e-05 | 39 % (59 %) | 94 % | WP_155851264.1 |
| ORF-34 | 18648 | 21293 | 881 | 98.7 | 6.03 | RNA polymerase | [Vibriophage VP4] | 0 | 73 %（84 %） | 99 % | YP_249577.1 |
| ORF-35 | 21559 | 21771 | 70 | 8.35 | 9.21 | hypothetical protein | [Aeromonas phage T7-Ah] | 7e-23 | 91 %  (94 %) | 95 % | QOC54764.1 |
| ORF-36 | 21786 | 21983 | 65 | 7.5 | 9.56 | hypothetical protein | [Aeromonas phage T7-Ah] | 2e-19 | 65 %  (72 %) | 100 % | QOC54764.1 |
| ORF-37 | 22057 | 22575 | 172 | 19.94 | 3.87 | hypothetical protein | [Stenotrophomonas phage IME15] | 8e-33 | 76 %  (86 %) | 96 % | YP_006990205.1 |
| ORF-38 | 22670 | 22861 | 63 | 7.45 | 5.61 | hypothetical protein | [Pectobacterium phage PP99] | 1e-04 | 37 %  (55 %) | 93 % | APW79695.1 |
| ORF-39 | 22881 | 23015 | 44 | 5.15 | 10.9 | hypothetical protein | [Stenotrophomonas phage IME15] | 3e-18 | 82 %  (90 %) | 100 % | YP_006990204.1 |
| ORF-40 | 23025 | 23234 | 69 | 7.78 | 9.8 | hypothetical protein | [Aeromonas phage T7-Ah] | 4e-39 | 96 %  (100 %) | 100 % | QOC54764.1 |
| ORF-41 | 24376 | 26124 | 582 | 66.1 | 5.44 | hypothetical protein | [Vibrio phage Rostov-1] | 0 | 79 %  (87 %) | 99 % | AVH85450.1 |
| ORF-42 | 26294 | 26449 | 51 | 5.32 | 5.75 | hypothetical protein | [Aeromonas phage T7-Ah] | 6e-25 | 96 %  (98 %) | 100 % | QOC54764.1 |
| ORF-43 | 26446 | 26586 | 46 | 4.74 | 5.97 | hypothetical protein | [Vibrio phage ICP3_2009_B] | 1e-5 | 63 %  （67 %） | 93 % | ADX87537.1 |
| ORF-44 | 26610 | 26849 | 79 | 8.39 | 4.85 | DNA packaging/maturation protein A | [Vibrio phage N4] | 1e-33 | 82 %  (91 %) | 89 % | YP_003347943.1 |
| ORF-45 | 26859 | 27797 | 312 | 33.71 | 5.29 | hypothetical protein | [Escherichia phage ECBP5] | 2e-22 | 38 %  (58 %) | 47 % | YP_009146420.1 |
| ORF-46 | 27797 | 28000 | 67 | 74.44 | 6.09 | holin | [Vibrio phage JSF25] | 2e-16 | 61 %  (76 %) | 95 % | ASU01128.1 |
| ORF-47 | 28018 | 29739 | 573 | 62.4 | 5.5 | phage tail fibers protein | [Stenotrophomonas phage IME15] | 6e-172 | 52 %  (66 %) | 97 % | YP_006990241.1 |
| ORF-48 | 29802 | 33926 | 1374 | 149.96 | 5.31 | protein inside capsid D | [Vibrio phage JSF36] | 2e-144 | 34 % (54 %) | 75 % | ASV43136.1 |
| ORF-49 | 33942 | 36035 | 697 | 77.89 | 5.55 | internal virion protein C | [Vibrio phage N4] | 2e-94 | 32 %  (52 %) | 94 % | AFH14432.1 |
| ORF-50 | 36107 | 36667 | 186 | 20.33 | 6.34 | internal virion protein B | [Vibrio phage ICP3_2009_A] | 5e-64 | 58 % (70 %) | 98 % | ADX87578.1 |
| ORF-51 | 36678 | 37124 | 148 | 17.13 | 6.13 | internal virion protein A | [Escherichia phage Penshu1] | 4e-11 | 35 % (51 %) | 85 % | QEG09812.1 |
| ORF-52 | 37117 | 37752 | 211 | 24.45 | 4.68 | Putative deoxyribonucleoside kinase | [Vibriophage VP4] | 4e-99 | 66 %  (80 %) | 98 % | YP_418244.1 |
| ORF-53 | 37831 | 40224 | 797 | 88.43 | 5.09 | tail tubular protein B | [Vibrio phage JSF31] | 0 | 67 % (79%) | 100 % | ASV42963.1 |
| ORF-54 | 40235 | 40828 | 197 | 22.31 | 4.26 | tail tubular protein A | [Vibrio phage N4] | 2e-94 | 70 %  (82 %) | 96 % | YP_003347933.1 |
