# Supplementary figures and images for: Lytic Bacteriophage PZL-Ah152 as Biocontrol Measures Against Lethal Aeromonas hydrophila Without Distorting Gut Microbiota
Source: Front Microbiol. 2022 Jul 12;13:898961. doi: 10.3389/fmicb.2022.898961 (PMC9315158; doi:10.3389/fmicb.2022.898961)

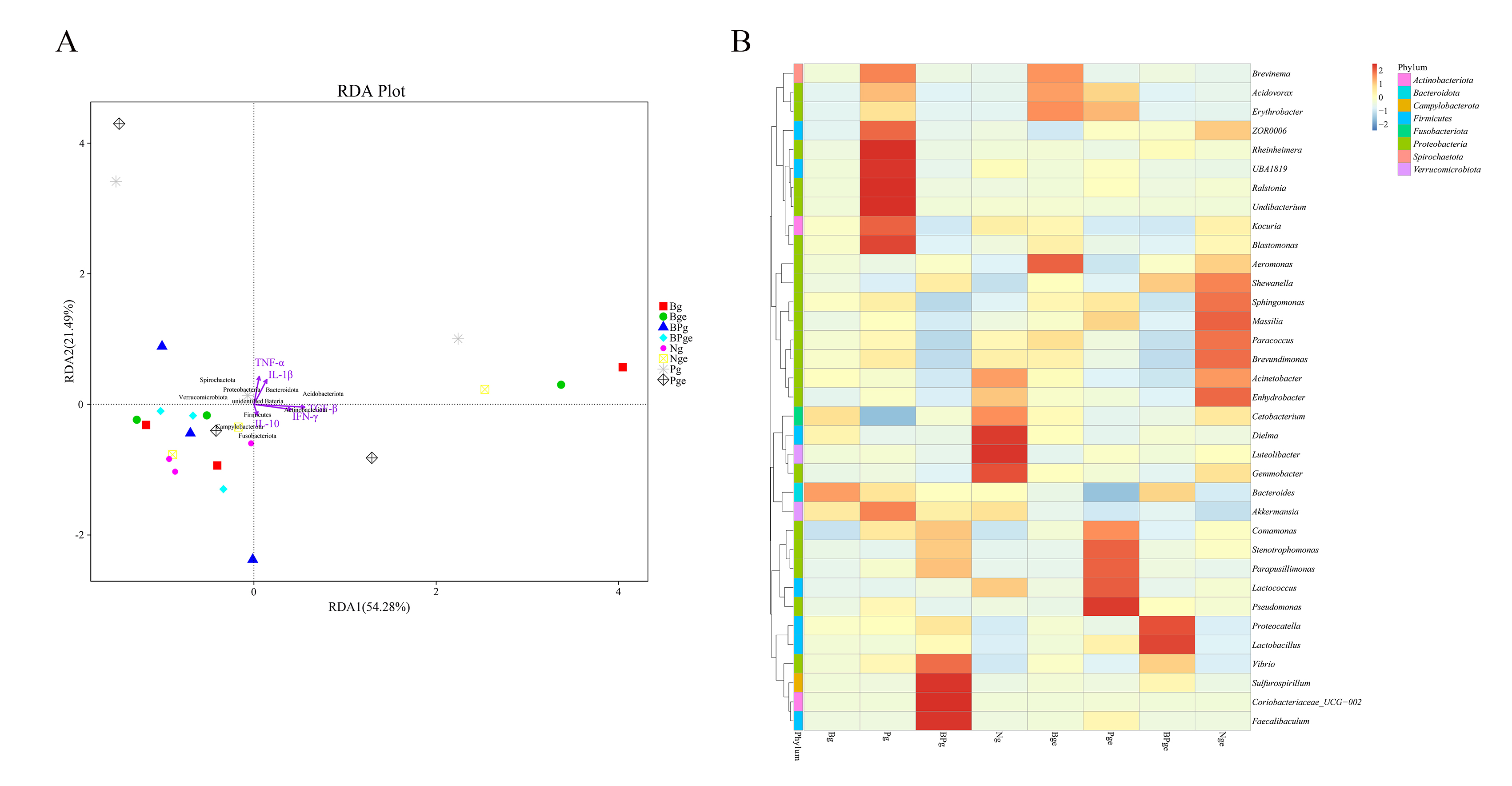

Supplement: Supplementary Figure 1 — The effects of A. hydrophila challenge and phage therapy on the composition of the gut microbiota. (A) Redundancy analysis (RDA) revealed the relationship between inflammatory factors and Intestinal flora in different groups. (B) Heatmapping shows that the abundance of intestinal bacteria at the genus level was significantly altered by treatment with phage PZL-Ah152 in challenged and normal fish. [file Image_1.JPEG]
